# Growth curve responses of two ECC-approved genotypes to locomotor, thermal, and nutritional challenges

**DOI:** 10.1016/j.psj.2026.107691

**Published:** 2026-08-22

**Authors:** Simona Mattioli, Marco Zampiga, Laura Menchetti, Cesare Castellini, Alessandro Dal Bosco, Luigia Bosa, Francesca Di Federico, Federico Sirri, Massimiliano Petracci, Fabrizio Pirrone, Rina Verdiglione, Marco Birolo

**Affiliations:** a Department of Agricultural, Environmental and Food Science, University of Perugia, 06121 Perugia, Italy; b Department of Agronomy, Food, Natural Resources, Animal and Environment (DAFNAE), University of Padova, 35020 Legnaro, Padova, Italy; c Department of Agricultural and Food Sciences, Alma Mater Studiorum - University of Bologna, 40064 Ozzano dell’Emilia, Bologna, Italy; d School of Biosciences and Veterinary Medicine, University of Camerino, 62024 Matelica, Macerata, Italy

**Keywords:** Broiler chickens, Growth plasticity, Gompertz model, Heat stress, Low-protein diet

## Abstract

Growing public concern for animal welfare is shifting European market demand towards alternative poultry production. A key challenge remains choosing a suitable genotype that balances improved welfare with productive performance and contributes to greater stability of production under challenging conditions. In this regard, three trials were performed to test the responses of medium-growing (MG; Ranger Gold) and slow-growing (SG; Kabir) genotypes to walking activity (TRIAL 1; Sw: spontaneous walking activity, Iw: induced walking activity for 30 min/day starting from 15 d of age), temperature (TRIAL 2; TN: thermoneutral at 22.8 ± 2.8°C; HS: heat stress at 27.2 ± 2.8°C), and dietary protein variation (TRIAL 3; CON: conventional basal diet; LOW: diet with a 10% reduction in the crude protein–to–metabolizable energy ratio), with respect to growth curve dynamics.

The Gompertz model and generalized linear mixed models were employed to analyze the growth curve of the animals. The parameters evaluated were A, adult body weight; B, the relative distance between initial body weight and projected asymptotic weight; k, the instantaneous relative growth rate; MGR, maximum growth rate; T50, T70 and T99 the estimated times required to reach 50%, 70%, and 99% of projected asymptotic body weight, respectively. The effects analysed were genotype, challenge, and their interaction. In each trial, genotype had a significant effect on all evaluated parameters, with MG outperforming SG across all model estimates. Regarding the genotype × treatment interaction, in TRIAL 1, the MG genotype exhibited higher values for B and MGR. In TRIAL 2, the increase in temperature decreased A and B only in MG. Significant effects of temperature and/or its interaction with genotype were also observed for T50 and MGR, with SG showing smaller changes in growth-curve parameters. In TRIAL 3, the LOW diet tended to reduce MGR, with a numerically larger reduction in MG birds, whereas SG birds showed only a limited change. In conclusion, new sustainable rearing strategies should account for the lower expected performance of SG strains while seeking to maximise the stability and overall value of the production system.

## Introduction

Growing public concern for animal welfare is shifting market demand toward alternative systems and genotypes, with clear implications for management and costs (Nassar, 2026). Until recently, EU guidance recommended slower-growing (SG) genotypes for organic and free-range systems, which represent roughly 5–6% of total poultry production (Fiorilla et al., 2023; Di Federico et al., 2026; Nompleggio et al., 2026). At the same time, the use of FG genotypes in intensive systems has attracted criticism because selection for rapid growth is consistently associated with higher risks of locomotor disorders, inactivity, and other welfare impairments (Tuyttens et al., 2008). Surveys indicate strong public support for higher-welfare standards, and several higher-welfare schemes now use breed-approval protocols to evaluate genotypes under standardised conditions at comparable body weights (Riuzzi et al., 2025). These protocols treat genotype as a central determinant of system robustness and welfare outcomes (Riber and Wurtz, 2024).

The European Chicken Commitment (ECC) promotes the adoption of broiler genotypes that meet specific welfare requirements, including limitations on growth rate and stocking density. However, ECC-approved genotypes encompass birds with different productive potential and developmental patterns. These differences may influence not only performance under standard conditions but also the stability of growth trajectories when birds are exposed to environmental, nutritional, or management challenges. Importantly, productive efficiency and stability of growth performance under challenging conditions represent partially independent dimensions of genotype response. In this context, growth-curve plasticity refers to changes in the shape, timing, or intensity of the modelled growth trajectory in response to environmental, nutritional, or management conditions.

Genetic selection for rapid growth has altered developmental patterns and the response of chickens to environmental and nutritional constraints (Rauw et al., 1998; Nicol et al., 2024). Resilience can be defined as the capacity of an animal or production system to maintain or recover biological function following exposure to a perturbation. Because resilience encompasses performance, survival, health, physiological, and behavioural responses, no single growth-related variable can provide a comprehensive assessment. Consequently, the evaluation of ECC-approved genotypes may benefit from considering not only average performance but also the stability and shape of their growth trajectories under different rearing conditions. In the present study, the stability of Gompertz growth parameters is therefore considered a performance-based component of the response to challenge.

Despite growing interest in ECC-aligned production, no studies have specifically compared the growth-curve plasticity of these ECC-approved genotypes under locomotor, thermal, and nutritional challenges. Quantifying these responses is important for productivity forecasting and for characterising genotype-specific performance patterns that inform genotype selection and management in higher-welfare systems (Menchetti et al., 2020; Mancinelli et al., 2023a). From a system perspective, genotypes that maintain performance under moderate nutrient dilution or environmental challenge offer greater flexibility for implementing lower-input feeding strategies without disproportionate productivity losses.

Growth-curve modelling provides a quantitative framework for investigating changes in growth trajectories. Unlike single end-point measures, such as final body weight, mathematical growth models estimate biologically interpretable parameters, including asymptotic weight (A), maturation rate (k), and maximum growth rate (MGR), thereby describing aspects of growth timing and intensity that are not captured by average daily gain alone (Aggrey, 2002; Rizzi et al., 2013). We hypothesised that ECC-approved genotypes would differ in their growth-curve responses to imposed locomotor, thermal, and nutritional challenges, resulting in genotype-specific changes in growth timing and intensity.

The three experimental trials generated complementary datasets that have been reported in separate companion studies. Those studies focus on productive performance, feed efficiency, behaviour, welfare, physiological responses, carcass traits, or meat quality. The present analysis addresses a distinct objective by modelling the complete longitudinal body-weight records to quantify genotype-specific changes in growth-curve shape, timing, and peak growth intensity.

This study evaluates the growth-curve responses of two ECC-approved broiler genotypes to three challenges: walking activity, heat stress, and dietary protein reduction.

## Materials and methods

### Ethics statement

All methods are reported following ARRIVE guidelines for the reporting of animal experiments. This study was carried out in accordance with the Guiding Principles in the Use of Animals for Scientific Purposes. It was approved by the Animal Bioethics Monitoring Committee of the University of Perugia (Walking activity trial: authorisation n° 79751 of 02/22/2024, protocol n. 10/2024), University of Padova (Heat stress trial: project 49/2024; Prot. n. 100806) and University of Bologna (nutritional trial: authorisation n° 4716/2024, Prot. n. 137903/2024).

### Animals, facilities, and experimental design

Three *in vivo* trials (TRIAL 1: walking activity; TRIAL 2: heat stress; TRIAL 3: low protein diet) were performed at the poultry farm of the Department of Agricultural, Environmental and Food Science, University of Perugia (TRIAL 1), Department of Agronomy, Food, Natural resources, Animals and Environment of the University of Padova (TRIAL 2), and Department of Agricultural and Food Sciences, University of Bologna (TRIAL 3), from February 2023 to May 2023.

Two ECC-approved genotypes with different growth rates were tested: a slow-growing genotype (SG; Kabir), characterised by a growth rate of 30–40 g/day, and a medium-growing genotype (MG; Ranger Gold), characterised by a growth rate of 40–50 g/day.

All chicks were obtained from the same commercial hatchery (Avizoo, Gruppo Pollarini, Via Emilia, Km 17 47020 Longiano, FC – Italy). One-day-old male chicks were vaccinated against coccidiosis, Marek’s disease, infectious bronchitis, and Newcastle disease at the hatchery, and transported to the experimental facilities by road in an authorised commercial poultry transport vehicle to the experimental poultry facilities of the University of Perugia, Padova and Bologna.

Birds were weighed on the day of their arrival and then once a week until commercial slaughter.

Mortality and pen feed consumption were measured daily during the trials. All birds had free access to water and feed.

Birds were slaughtered at different ages (50 to 56 days of age for MG; 70 to 81 days for SG) according to the commercial procedures commonly used for MG and SG chicken production.

### Trial 1 – walking activity

A total of 200 birds were divided into two experimental groups and housed in 3 × 3 m indoor floor pens (25 birds per pen, four pens per genotype, <30 kg/m^2^ stocking density). Each pen was provided with one feeder and 3 drinkers, and the floor was covered with wood-shaving litter. Birds were assigned to either a control group, in which walking activity was spontaneous (Sw), or an induced-walking group (Iw), in which operator-supervised locomotor activity was performed daily for 30 min at approximately 4 km/h, starting at 15 d of age (Mattioli et al., 2021). Before the activity period, birds were allowed approximately 10 min to adapt to the presence of the operators. Operators then walked behind the birds around the paddock to encourage movement. Completion of each scheduled exercise session was monitored by the operators. Behavioural responses to the treatment were assessed separately and are reported in Nompleggio et al. (2026). The room temperature was initially set at 30–32°C and then reduced by 1-2°C every 3 d until approximately 20°C was reached; this temperature was maintained until the end of the trial. The photoperiod was set to 16 h light and 8 h dark. Birds had *ad libitum* access to feed and water. Individual body weight was recorded at placement and weekly thereafter, whereas pen feed consumption and mortality were recorded daily.

The birds were fed according to a commercial three-phase feeding program: starter diet from 1 to 15 d of age; grower diet from 16 to 31 d of age; finisher diet from 32 d of age until slaughter.

The starter, grower, and finisher diets contained 24.77%, 21.87%, and 20.51% protein, and 4.52%, 3.73%, and 3.84% lipids, respectively. Detailed ingredient composition and chemical analysis are available in Di Federico et al. (2026). At 12 days of age 3 birds for pens were removed for specific study on muscle development, then 22 birds per pen were used for live weight evaluations.

The genotypes were slaughtered upon reaching their respective commercial weights: at 63 d of age for MG and 81 d of age for SG.

### Trial 2 – heat stress

The study was conducted at the poultry facility of the University of Padova, which comprised two identical rooms equipped with radiant ceiling heating, cooling, controlled ventilation, and a programmable lighting system. To simulate natural conditions, a dawn-dusk effect was implemented via a gradual 15-minute transition in light intensity. The lighting schedule provided 24 hours of continuous light for the first two days post-arrival; from day 3 to day 12, the dark period was incrementally increased to six consecutive hours and maintained until the conclusion of the trial.

A total of 480 one-day-old male chicks (240 MG and 240 SG) were distributed among 24 pens (12 pens per genotype). Within each genotype, half of the pens were located in the room maintained under thermoneutral (control) conditions, while the remaining half were subjected to heat stress. Each room contained 12 floor pens measuring 2.60 × 1.25 m, with a total floor area of 3.25 m² per pen. Each pen housed 20 birds and was equipped with five automatic nipple drinkers fitted with drip-saving cups and one manual circular feeder. The concrete floor was covered with approximately 15 cm of wood-shaving litter, corresponding to about 5 kg/m². Stocking density was maintained at or below 30 kg live weight/m² throughout the trial. In both rooms, the initial ambient temperature was set at 30°C for the first three days. Subsequently, the temperature in the control room was gradually decreased to 20-22°C by the fifth week, following Aviagen (2018) recommendations. Conversely, in the heat stress room, the temperature was reduced from 30°C to 26-27°C and held constant until slaughter. On average, the mean 24-h temperature in the heat stress room was 4.4°C higher than in the control room. This temperature regime was designed to reproduce a chronic, moderate thermal challenge representative of prolonged exposure to elevated ambient temperatures during the warm season in commercial broiler production.

Environmental conditions were continuously recorded using a data logger (P5185, PeakTech, Prüfund Messtechnik GmbH Gerstenstieg, Ahrensburg, Germany) positioned 30 cm above the floor in the center of each room. Since the two temperature conditions were each applied to one environmentally controlled room, temperature and room effects could not be separated. Accordingly, temperature effects should be interpreted as differences observed between the two environmental conditions established in the rooms used in this trial.

Throughout the trial, chickens were fed three corn-soybean meal-based diets: a starter diet (days 0-13; 20.8% CP, 5.00% EE, 2.36% CF), a grower diet (days 14-28; 18.6% CP, 5.12% EE, 2.45% CF) in crumbled form, and a finisher diet (day 29 to slaughter; 17.7% CP, 4.97% EE, 2.42% CF) in pelleted form. All diets were produced by a commercial feed mill (Consorzio Agrario di Treviso e Belluno, Italy). Commercial slaughter occurred at 55 days of age for MG and 76 days of age for SG. Further details regarding ingredient composition, chemical analyses, and housing conditions are available in Pontalti et al. (2025).

### Trial 3 – low protein diet

A total of 1,200 birds (600 birds/genotype) were randomly divided into four experimental groups according to genotype (MG and SG) and dietary treatment (CON: conventional basal diet for fast-growing broiler chickens; and LOW: diet with 10% reduced crude protein-to-metabolizable energy ratio). Each experimental group comprised six pens, with the concrete floor covered with bedding material (i.e., pelleted straw; 3 − 4 kg/m^2^). Each pen was equipped with two circular pan feeders (providing 2 cm of front space per bird) and an independent drinking system with eight nipple-type drinkers. According to the ECC protocol, the stocking density was consistently maintained below 30 kg of live weight per m². Photoperiod was managed according to the EU legislation (Directive 2007/43/EC): 23 h light and 1 h dark were provided from 0 to 7 d, while 18 h light and 6 h dark were used for the remaining period. Environmental temperature and humidity were defined throughout the trial following the standard recommendations for broiler chickens, with adjustments made according to bird age. The room temperature was initially set at 30°C and then reduced by 1-2°C every 3 d until approximately 20°C was reached; this temperature was maintained until the end of the trial. Two experimental diets were administered following a three-phase feeding program: starter, 0–13 d; grower, 14–27 d; finisher, from 28 d until slaughter age. The CON diet was formulated according to the nutritional recommendations for fast-growing broilers. Conversely, the LOW diet was characterized by a 10% reduction in the crude protein content and the same metabolizable energy as the CON diet. In detail, the CON and LOW diets contained a crude protein content of 22.5% and 20.3% in the starter phase, 20.5% and 18.3% in the grower phase, and 18.5% and 16.5% in the finisher phase, respectively. Crude protein was reduced in the LOW diets while maintaining the same amino acid profile as in the CON diets. The metabolizable energy contents were 3,015, 3,070, and 3,100 kcal/kg for both CON and LOW in the starter, grower, and finisher phases. The feed physical form was crumbled during the starter phase and then pelleted. MG and SG birds were slaughtered in a commercial processing plant at 50 and 77 days of age.

### Statistical analysis

The Gompertz model (González Ariza et al., 2021; Mancinelli et al., 2023a; Menchetti et al., 2024) was employed to analyze the growth curve of the animals using the following equation:

Y = A*EXP(-B*EXP(-k*t)) where Y represents body weight (BW) at age t, and A denotes the asymptotic or adult body weight. Parameters B and k describe the shape and temporal development of the curve. B is a dimensionless integration constant related to the ratio between the asymptotic and initial body weights and contributing to the temporal position of the growth curve. Parameter k represents the instantaneous relative growth rate, with higher values indicating earlier maturation. Additional biologically meaningful parameters were derived from the model. These included the time required to reach 50, 70, and 99% of adult body weight (T50, T70, and T99, respectively), and the maximum growth rate (MGR), corresponding to the growth rate at the inflection point (IP; i.e., the age at which growth rate peaks), was calculated as MGR = (A/e) × k (Mancinelli et al., 2023b; Menchetti et al., 2024). Because A represents the model-estimated asymptotic weight and birds were slaughtered at genotype-specific commercial ages, this parameter and the related derived parameters (T50, T70, and T99) should be interpreted with caution, as their estimates may extend beyond the observed growth period. Within each genotype and trial, all treatment groups were monitored for the same duration. In Trials 1 and 2, individual Gompertz curves were estimated only for birds with complete longitudinal body-weight records. In Trial 3, growth curves were fitted to pen-level body-weight data, while mortality was recorded separately for each pen. Each trial was analysed separately, and no statistical comparisons of absolute growth-curve parameter estimates were performed among trials. Data distribution was evaluated using diagnostic plots to identify potential outliers. Based on this screening, all data from one animal were excluded due to multiple parameters being classified as extreme outliers; additionally, one outlying value for parameter A was removed from the dataset. For graphical representation, a representative growth curve for each experimental group and genotype was generated using the mean parameter values obtained from the individual fitted curves. Conversely, the parameter estimates obtained from each individual curve were retained as individual observations and used in the subsequent inferential analyses using generalized estimating equations (GEE), with pen specified as the subject effect to account for the clustering of birds within pens. Thus, in Trials 1 and 2, the individual bird was the analytical unit, whereas the pen was the experimental unit. An exchangeable working correlation structure was assumed for observations within the same pen. In Trial 3, growth curves were fitted to pen-level body-weight data, while mortality was recorded separately for each pen. For each experiment, the model evaluated the fixed effects of challenge condition, genotype, and their interaction. Various distributions and link functions were tested and evaluated based on the Akaike Information Criterion (AIC) to determine the best model fit. Ultimately, a normal distribution with an identity link function was used for parameters B, T50, T70, and T99, while the gamma distribution with a log link was selected for A, k, and MGR. Results are expressed as estimated marginal means with 95% Wald confidence intervals (95% CI) to facilitate the interpretation of the magnitude and precision of the estimated effects. When the genotype × challenge interaction was significant, pairwise comparisons were performed among the estimated marginal means of the four genotype × challenge combinations using the Bonferroni adjustment for multiple comparisons.

GraphPad Prism, version 8.0 (GraphPad Software, San Diego, CA) was used to plot the growth curves, while SPSS Statistics version 25 (IBM, SPSS Inc., Chicago, IL) was used to analyze the parameters. The level of statistical significance was set at P < 0.05. Values of 0.05 ≤ P < 0.10 were considered indicative of a statistical tendency.

## Results

Mortality remained low across the three trials, reaching on average 1.5% in Trial 1, 2.2% in Trial 2, and 1.3% in Trial 3, without significant genotype, treatment, or genotype × treatment effects (data not shown in table).

**Fig. 1:**
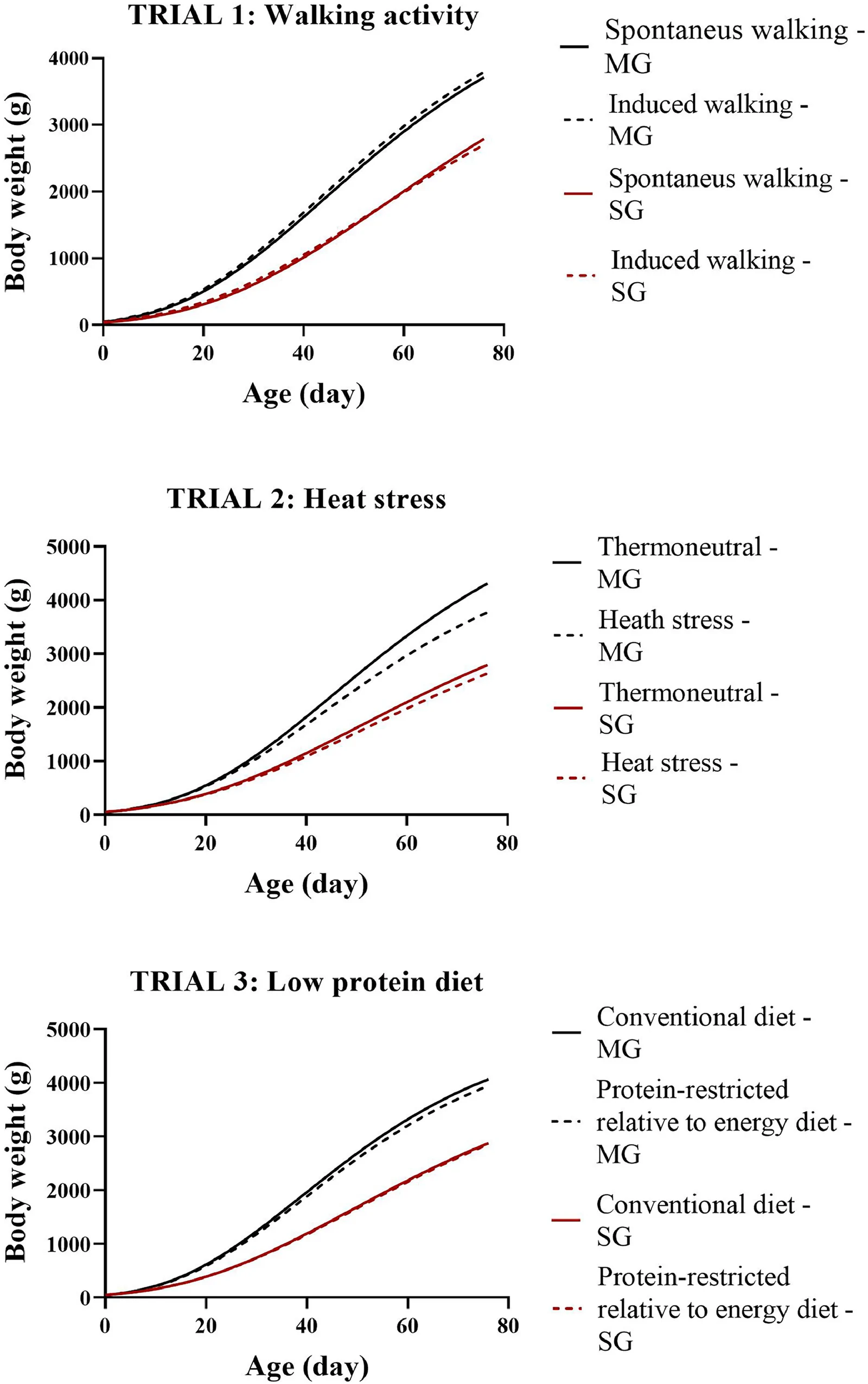
Growth curves of the genotypes under three experimental challenges. Walking activity (panel A), heat stress (panel B), and dietary protein content variation (panel C). Representative growth curves for each experimental group were generated using the mean values of the Gompertz parameters estimated from the individual fitted curves. MG = medium-growing and SG = slow-growing genotypes.

Estimated marginal means, 95% Wald confidence intervals, model results, and pairwise comparisons for the fitted and derived growth-curve parameters are presented in Table 1, Table 2, Table 3. Fig. 1 shows the graphical representation of the growth curves of the genotypes in the three experiments.

**Table 1:** Estimated marginal means (95% Wald confidence intervals), statistical model results, and multiple comparisons for Gompertz growth curve parameters and derived indices in medium-growing (MG) and slow-growing (SG) broiler genotypes exposed to spontaneous or induced walking activity. The analysis included 176 animals housed in 8 pens, with 22 animals per pen and 44 animals in each movement × genotype combination. Four pens were originally assigned to each temperature × genotype combination. The pen was considered the experimental unit (n = 8 pens per movement × genotype combination).

Parameter	Spontaneous walking		Induced walking		P-value		
	MG	SG	MG	SG	M	G	M × G
A (g)	5,173^a^ (5095–5253)	5,072^ab^ (4622–5565)	5,226^a^ (5203–5248)	4,627^b^ (4619–4635)	0.089	0.003	0.034
B	4.66^c^ (4.65–4.67)	4.84^d^ (4.75–4.93)	4.58^b^ (4.57–4.58)	4.55^a^ (4.54–4.55)	<0.001	0.001	<0.001
k	0.035 (0.035–0.035)	0.028 (0.025–0.030)	0.035 (0.035–0.035)	0.028 (0.028–0.028)	0.566	<0.001	0.765
T50 (days)	56.3 (55.9–56.7)	71.5 (65.4–77.6)	55.7 (55.5–55.8)	68.5 (68.4–68.7)	0.253	<0.001	0.449
T70 (days)	76.0 (75.5–76.5)	97.1 (90.3–103.9)	75.3 (75.1–75.6)	92.8 (92.7–93.0)	0.164	<0.001	0.300
T99 (days)	181.6 (180.6–182.7)	230.2 (213.1–247.2)	180.9 (180.4–181.4)	223.3 (222.9–223.7)	0.386	<0.001	0.481
MGR (g/d)	63.6^c^ (63.2–64.0)	49.6^b^ (48.6–50.7)	64.2^d^ (64.1–64.2)	46.3^a^ (46.3–46.3)	<0.001	<0.001	<0.001

^a, b, c^ Different lowercase superscript letters within a row indicate significant differences among M × G combinations based on pairwise comparisons with Bonferroni adjustment (P ≤ 0.05). MG: medium-growing chickens; SG: slow-growing chickens; M: movement; G: genotype; M × G: movement × genotype; A: projected asymptotic body weight; B: parameter representing the relative distance between initial body weight and projected asymptotic weight; k: the instantaneous relative growth rate; T50, T70 and T99: estimated times required to reach 50%, 70%, and 99% of projected asymptotic body weight, respectively; MGR: maximum growth rate.

**Table 2:** Estimated marginal means (95% Wald confidence intervals), statistical model results, and multiple comparisons for Gompertz growth curve parameters and derived indices in medium-growing (MG) and slow-growing (SG) broiler genotypes exposed to thermoneutral or heat stress conditions. The analysis included 421 animals housed in 24 pens, with 15 to 19 animals per pen after data exclusions. Six pens were originally assigned to each temperature × genotype combination. The pen was considered the experimental unit.

Parameter	Thermoneutral		Heat stress		P-value		
	MG	SG	MG	SG	T	G	T × G
A (g)	6,083^d^ (5,939–6,232)	4,501^b^ (4,397–4,608)	5,163^c^ (4,928–5,410)	4,309^a^ (4,240–4,378)	<0.001	<0.001	<0.001
B	4.835^d^ (4.765–4.906)	4.385^b^ (4.374–4.397)	4.621^c^ (4.597–4.646)	4.304^a^ (4.268–4.339)	<0.001	<0.001	0.002
k	0.035^b^ (0.034–0.036)	0.029^a^ (0.029–0.029)	0.035^b^ (0.035–0.036)	0.029^a^ (0.028–0.029)	0.797	<0.001	0.047
T50 (days)	56.6^a^ (55.7–57.5)	63.5^b^ (62.8–64.3)	54.7^a^ (53.5–55.9)	64.5^b^ (63.5–65.6)	0.348	<0.001	0.004
T70 (days)	76.0^a^ (74.7–77.3)	86.4^b^ (85.4–87.4)	73.9^a^ (72.4–75.4)	88.1^b^ (86.5–89.4)	0.683	<0.001	0.006
T99 (days)	180.1^a^ (176.8–183.4)	209.4^b^ (207.0–211.7)	176.9^a^ (173.4–180.3)	214.1^b^ (210.5–217.7)	0.644	<0.001	0.015
MGR (g/d)	76.6^d^ (75.4–77.9)	49.6^b^ (48.6–50.7)	65.6^c^ (63.6–67.6)	46.3^a^ (46.3–46.3)	<0.001	<0.001	<0.001

^a, b, c, d^ Different lowercase superscript letters within a row indicate significant differences among T × G combinations based on pairwise comparisons with Bonferroni adjustment (P ≤ 0.05). MG: medium-growing chickens; SG: slow-growing chickens; T: temperature; G: genotype; T × G: temperature × genotype; A: projected asymptotic body weight; B: parameter representing the relative distance between initial body weight and projected asymptotic weight; k: the instantaneous relative growth rate; T50, T70 and T99: estimated times required to reach 50%, 70%, and 99% of projected asymptotic body weight, respectively; MGR: maximum growth rate.

**Table 3:** Estimated marginal means (95% Wald confidence intervals), statistical model results, and multiple comparisons for Gompertz growth curve parameters and derived indices in medium-growing (MG) and slow-growing (SG) broiler genotypes fed a control or a low-input diet (10% reduced crude protein-to-metabolizable energy ratio). The analysis included 24 pens, with one observation per pen and no data exclusions. The pen was considered both the observational and experimental unit (n = 24 pens).

Parameter	Control diet		Low-protein diet		P-value		
	MG	SG	MG	SG	D	G	D × G
A (g)	5,110 (4,864–5,369)	4,445 (4,395–4,496)	5,006 (4,816–5,204)	4,490 (4,449–4,532)	0.750	<0.001	0.343
B	4.685 (4.634–4.736)	4.491 (4.469–4.512)	4.670 (4.638–4.701)	4.464 (4.424–4.504)	0.269	<0.001	0.766
k	0.040 (0.039–0.041)	0.031 (0.030–0.031)	0.039 (0.038–0.040)	0.030 (0.030–0.031)	0.121	<0.001	0.864
T50 (days)	48.1 (46.7–49.5)	60.8 (60.1–61.6)	48.8 (47.4–50.2)	61.8 (61.0–62.7)	0.136	<0.001	0.783
T70 (days)	64.8 (63.0–66.6)	82.4 (81.4–83.5)	65.8 (63.8–67.7)	83.9 (82.7–85.1)	0.120	<0.001	0.760
T99 (days)	154.6 (150.6–158.6)	198.6 (196.1–201.2)	157.1 (152.3–161.9)	202.4 (199.2–205.7)	0.099	<0.001	0.726
MGR (g/d)	74.7 (72.5–76.9)	50.2 (49.9–50.6)	72.0 (71.1–72.8)	49.8 (49.1–50.5)	0.099	<0.001	0.128

MG: medium-growing chickens; SG: slow-growing chickens; D: diet; G: genotype; D × G: diet × genotype interaction; A: projected asymptotic body weight; B: parameter representing the relative distance between initial body weight and projected asymptotic weight; k: the instantaneous relative growth rate; T50, T70 and T99: estimated times required to reach 50%, 70%, and 99% of projected asymptotic body weight, respectively; MGR: maximum growth rate.

The Gompertz model provided a good fit to the growth data in all trials. Across experimental groups, R² values ranged from 0.982 to 1.000, whereas RMSE values ranged from 2.9 to 139.4 g. N

Group-specific R² and RMSE ranges are reported in Supplementary Table S1.

### Trial 1 – effect of walking activity

Genotype significantly affected A, B, k, T50, T70, T99, and MGR, with MG birds generally showing a higher growth rate and earlier attainment of the selected proportions of projected A than SG birds (Table 1).

Induced walking activity affected only parameters A, B, and MGR, with significant movement × genotype interactions. Overall, movement significantly reduced B (on average: 4.75 vs. 4.56 for SW and IW, respectively; −3.96%) and MGR (56.6 vs. 55.2 g/d; −2.43%). Notably, the effect of induced walking activity was not significant in the MG genotype, whereas in SG birds it reduced A from 5,072 to 4,627 g, B from 4.84 to 4.55, and MGR from 49.6 to 46.3 g/day (Table 1). No significant effects of walking activity or movement × genotype interactions were detected for k, T50, T70, or T99.

### Trial 2 – effect of heat stress

Genotype significantly affected all fitted and derived growth-curve parameters (P < 0.001), with MG birds showing higher projected A, k, and MGR and earlier attainment of 50%, 70%, and 99% of projected A than SG birds (Table 2).

Heat stress significantly reduced A, B, and MGR (P < 0.001). Significant temperature × genotype interactions were observed for A (P < 0.001), B (P = 0.002), k (P = 0.047), T50 (P = 0.004), T70 (P = 0.006), T99 (P = 0.015), and MGR (P < 0.001). The interaction for k approached significance (P = 0.054). Pairwise comparisons showed that heat stress reduced A in MG birds from 6,083 to 5,163 g and from 4,501 to 4,309 g in SG birds. Similarly, B decreased from 4.84 to 4.62 in MG birds and from 4.39 to 4.30 in SG birds. MGR decreased from 76.6 to 65.6 g/day in MG birds and from 49.6 to 46.3 g/day in SG birds. Although significant interactions were also detected for k, T50, T70, and T99, pairwise comparisons between thermal conditions within genotype did not identify significant changes (Fig. 1 and Table 2).

### Trial 3 – effect of low-protein diet

Genotype significantly affected all fitted and derived growth-curve parameters (P < 0.001), with MG birds showing higher A, k, and MGR and earlier estimated T50, T70 and T99 than SG birds (Table 3).

The low-protein diet tended to increase T99 (P = 0.099) and reduce MGR (P = 0.099). MGR decreased numerically from 62.5 g/day with the control diet to 60.9 g/day with the low-protein diet, while T99 increased from approximately 176.6 to 179.8 days. No significant diet effect was detected for A, B, k, T50, or T70, and no diet × genotype interaction was significant for any parameter (Table 3).

## Discussion

Slow-growing chickens are widely recognised as having lower feed efficiency and, consequently, potentially reduced environmental sustainability (Zampiga et al., 2021). While reduced growth intensity may impair feed efficiency under optimal conditions, it may also be associated with smaller changes in growth performance when birds face environmental, behavioural, or nutritional constraints (Khire and Ryba, 2025).

Within each independently analysed trial, the two ECC-approved strains differed in their growth responses to the imposed challenges. The slower-growing genotype showed smaller changes in several growth-curve parameters under some of the tested conditions, although the magnitude and direction of the response depended on the specific challenge. These findings indicate that baseline productive performance and stability of the growth trajectory under challenge are distinct components of genotype response.

A possible conceptual framework for interpreting these differential responses is resource allocation theory, which describes how organisms distribute limited energetic and nutritional resources among competing functions such as growth, maintenance, thermoregulation, and immune defence (Rauw et al., 1998; Klasing, 2007). Previous studies suggest that selection for rapid growth and high feed efficiency can shift resource use toward structural tissue deposition and may alter responses to environmental constraints. In the present study, this framework is used only as a possible interpretation of the genotype-specific growth-curve responses.

Within the Gompertz framework, these allocation patterns can be interpreted through biologically meaningful parameters. Asymptotic weight (A) represents the projected upper body-weight limit of the fitted curve, whereas k describes the relative rate of maturation, and the maximum growth rate (MGR), occurring at the inflection point, represents the peak intensity of structural growth. Under challenging conditions, reductions in MGR and/or A can therefore be interpreted as quantitative indications of reduced peak growth intensity and/or a lower projected asymptotic body weight.

In the present analytical framework, maintenance of A and MGR under challenge was interpreted as greater stability of the modelled growth trajectory, whereas pronounced reductions indicate greater sensitivity of growth performance to the tested condition.

The combination of declining energetic resources available for movement and a restricted speed range highlights how exercise performance is compromised in highly productive broilers. Tickle et al. (2018) demonstrated that activity levels and metabolic costs are inversely related: heavier birds tend to be relatively inactive, likely because locomotion is energetically expensive. Thus, in birds selected for rapid growth and large pectoral mass, the musculoskeletal and respiratory systems become progressively less capable of sustaining work.

The reduction in MGR observed in SG under the locomotor challenge should not be interpreted solely as an increased energetic cost of movement. At the flock level, higher activity is not necessarily associated with poorer feed efficiency, and associations between activity distribution and growth performance can be context-dependent (Donnelly et al., 2026). In our study, a more conservative interpretation is that imposed locomotor activity altered growth intensity in SG birds, as reflected by changes in the relative position of the growth curve and by the reduction in MGR, indicating greater sensitivity of peak growth dynamics to physical effort. The imposed exercise would be expected to increase the metabolic demand associated with locomotion, although the present growth-curve data do not quantify this demand or its effects on nutrient use. Thus, the present results are consistent with previous evidence that the relationship between locomotor activity and growth performance is genotype- and context-dependent rather than uniformly detrimental.

Within the allocation theory framework, genetic selection for higher growth intensity may contribute to genotype-specific responses when alternative physiological demands arise, although energy allocation was not directly measured in the present study. Genotypes with higher growth intensity may therefore have less scope for modulating peak growth in response to locomotor challenges. This suggests that imposed locomotor demand can reveal genotype-specific differences in growth-curve responses even among ECC-approved genotypes.

SG chickens generally show smaller performance alterations than MG or FG birds when exposed to thermal stress. Under elevated temperatures, SG birds often show lower physiological stress and smaller reductions in daily weight gain and final body weight (Lu et al., 2007; Soleimani et al., 2011; Perini et al., 2020; Huerta et al., 2023). Conversely, commercial hybrids frequently exhibit worsened feed efficiency under heat stress (Zhang et al., 2017; Goo et al., 2019). Our results partially align with this pattern: heat stress reduced projected asymptotic weight-related parameters and peak growth intensity in both genotypes, although the magnitude of these changes was greater in MG birds. The thermal regime was intended to represent a chronic, moderate challenge comparable to prolonged warm-season exposure in commercial production, and its biological effectiveness was confirmed by the reductions in feed intake and growth performance described by Pontalti et al. (2025). This indicates that the magnitude of the growth response to elevated temperature differs even among ECC-approved genotypes with relatively slow growth rates. Although direct physiological, behavioural, and molecular responses were measured in the same trial, they are analysed separately and are not integrated into the present growth-curve analysis. Therefore, the mechanisms underlying the genotype-specific growth responses cannot be established from the data presented here alone. A negative correlation between A and k has been previously reported (Mignon-Grasteau et al., 2000), illustrating how a rapid decline in growth rate after the inflection point can reduce asymptotic body weight.

Previous studies have attributed the lower sensitivity of some SG genotypes to elevated temperatures partly to their lower body weight and metabolic heat production (Soleimani et al., 2011; Gogoi et al., 2021). Intensive selection for growth and muscle development has been associated with reduced thermoregulatory capacity (Onagbesan et al., 2023), potentially contributing to the greater heat susceptibility of high-performing genotypes (Brugaletta et al., 2021). Overall, the larger changes observed in MG birds are consistent with previous studies reporting greater thermal sensitivity in genotypes characterised by higher growth intensity and metabolic heat production.

In the present study, the low-protein diet involved a 10% reduction in crude protein compared with the control diet. This moderate but biologically relevant restriction tended to reduce MGR, with a numerically larger reduction in MG birds, whereas SG birds maintained growth-curve parameters closer to those observed under the control diet. The primary effect concerned the inflection-point growth rate, whereas asymptotic weight and maturation-related parameters showed limited variation. These findings suggest that the 10% reduction in dietary protein content primarily affected the estimated peak growth rate at the inflection point, while having limited effects on the other features of the modelled growth trajectory. Although this pattern may be interpreted within a resource-allocation framework, the present analysis does not establish how nutrients were partitioned between maintenance and growth. As discussed above, genotypes with higher baseline growth intensity may be more sensitive to moderate nutrient dilution, exhibiting sharper reductions in peak growth rate than slower-growing counterparts.

This interpretation aligns with previous evidence showing that local or slower‑growing genotypes are generally less sensitive to suboptimal or low‑input diets than fast‑growing commercial hybrids (Perella et al., 2009). A similar genotype-dependent response to reduced nutrient density was previously reported by Menchetti et al. (2024), although that study involved genotypes with a broader range of growth rates. The present study included only ECC‑approved genotypes. Nevertheless, the contrasting responses of MG and SG suggest that the stability of growth performance under moderate protein reduction varies even within slower-growing categories. The numerical response observed here is therefore consistent with previous evidence that genotypes differing in growth potential may also differ in their response to moderate nutrient dilution, although the non-significant interaction in the present trial does not support a definitive genotype-specific effect.

The results of the independently analysed trials suggest that changes in peak growth intensity, represented by MGR, may be particularly informative when characterising genotype responses to different challenges. Changes in MGR describe variation in the estimated peak growth rate at the inflection point, whereas parameters such as A and k reflect projected asymptotic weight and maturation timing, respectively. Thus, MGR provides a sensitive metric for detecting changes in peak growth performance, although it does not identify the physiological mechanisms responsible for those changes.

Genotype-specific slaughter ages also resulted in different lengths of the observed growth period. Although treatment groups within each genotype were monitored until the same age, comparisons between genotypes should be interpreted cautiously because these parameters are model-derived projections and may be influenced by the amount of information available on the later portion of the growth curve.

Mortality was low and similarly distributed among experimental groups, suggesting that the exclusion of incomplete individual growth records was unlikely to substantially influence comparisons of growth-curve parameters. Nevertheless, the fitted curves describe the growth trajectories of birds with complete longitudinal records and should not be interpreted as a comprehensive assessment of resilience, which also encompasses survival, physiological and welfare outcomes. Moreover, the present analysis was specifically focused on individual growth trajectories and did not integrate all the complementary variables collected within the broader project, including feed intake, feed conversion ratio, metabolomic and welfare indicators. Behavioural observations were included in the walking-activity and heat-stress trials. Some of these outcomes have already been reported in companion studies (Nompleggio et al., 2026; Pontalti et al., 2025), while the remaining datasets are being analysed separately.

## Conclusion

The two ECC-approved genotypes differed in the stability of their growth trajectories under locomotor, thermal, and nutritional challenges. Maximum growth rate was among the most responsive model-derived parameters. Under heat stress, the slower-growing genotype showed smaller changes than the medium-growing genotype, whereas the low-protein diet produced only a tendency toward reduced MGR without a significant genotype-specific response.

These findings indicate that a genotype with higher productive efficiency under standard conditions does not necessarily show greater stability of growth performance when exposed to environmental or nutritional challenges. Within slower‑growing categories, genotypes can differ substantially in the magnitude of their growth response to environmental or nutritional challenges, while possible genotype-specific responses to moderate nutrient dilution require further investigation. Growth‑curve modelling therefore offers a quantitative framework to detect genotype‑specific stability patterns that are not apparent from end‑point measures of body weight or feed efficiency alone.

Future studies should integrate growth trajectories with physiological, behavioural, welfare, and feed-efficiency indicators to clarify the mechanisms underlying these responses and assess their practical relevance under commercial conditions.

## Author contribution

All authors (Simona Mattioli, Marco Zampiga, Laura Menchetti, Cesare Castellini, Alessandro Dal Bosco, Luigia Bosa, Francesca Di Federico, Federico Sirri, Massimiliano Petracci, Fabrizio Pirrone, Rina Verdiglione, Marco Birolo) contributed directly to the intellectual development of this project. Simona Mattioli, Marco Zampiga, Laura Menchetti, Marco Birolo, conceived the study design and formulated the core research hypotheses. Luigia Bosa, Francesca Di Federico, Fabrizio Pirrone, Rina Verdiglione developed the analytical framework and interpreted the findings. Simona Mattioli, Marco Zampiga, Laura Menchetti, Marco Birolo, and Cesare Castellini participated in drafting, Alessandro Dal Bosco, Federico Sirri, and Massimiliano Petracci critically revising, and finalizing the manuscript, providing essential intellectual input to the study.

## Declaration of generative AI and AI-assisted technologies in the manuscript preparation process

During the preparation of this work, the authors used Microsoft Copilot for English language editing and revision. The authors reviewed and edited the output as needed and take full responsibility for the content of the published article.

## Funding information

This research was funded by NextGenerationEU_ PRIN2022 CUP J53D23009690006 (Prot. 20228ANBKH).

## Disclosures

The authors declare the following financial interests/personal relationships which may be considered as potential competing interests:

Simona Mattioli reports financial support was provided by NextGenerationEU_ PRIN2022 CUP J53D23009690006. If there are other authors, they declare that they have no known competing financial interests or personal relationships that could have appeared to influence the work reported in this paper.

## Footnotes

Supplementary material associated with this article can be found, in the online version, at doi:10.1016/j.psj.2026.107691.
