# Supplementary material for: Growth curve responses of two ECC-approved genotypes to locomotor, thermal, and nutritional challenges
Source: Poult Sci. 2026 Aug 22;105(12):107691. doi: 10.1016/j.psj.2026.107691 (PMC13594876; doi:10.1016/j.psj.2026.107691)
Supplement: Supplementary file 1 [file mmc1.docx]

**Supplementary Table S1.** Goodness-of-fit statistics of the Gompertz growth model for each experimental group and genotype. R² and RMSE ranges obtained from the individual Gompertz curves fitted within each experimental group. In Trials 1 and 2, statistics refer to individual bird-level curves, whereas in Trial 3 they refer to pen-level curves. SW = spontaneous walking; IW = induced walking; TN = thermoneutral; HS = heat stress; CD = control diet; LP = low-protein diet; MG = medium-growing genotype; SG = slow-growing genotype.

| **Trial** | **Experimental challenge** | **Group** | **Genotype** | **R² range** | **RMSE range (g)** |
| --- | --- | --- | --- | --- | --- |
| 1 | Walking activity | SW | MG | 0.982–0.999 | 36.4–139.4 |
| 1 | Walking activity | SW | SG | 0.983–0.999 | 30.5–128.0 |
| 1 | Walking activity | IW | MG | 0.983–0.999 | 26.6–135.9 |
| 1 | Walking activity | IW | SG | 0.986–0.998 | 36.4–115.0 |
| 2 | Heat stress | TN | MG | 0.992–1.000 | 8.1–83.8 |
| 2 | Heat stress | TN | SG | 0.997–1.000 | 10.5-44.4 |
| 2 | Heat stress | HS | MG | 0.996–1.000 | 7.0–61.6 |
| 2 | Heat stress | HS | SG | 0.998–1.000 | 7.1–52.5 |
| 3 | Dietary protein | CD | MG | 0.999–1.000 | 2.9–11.4 |
| 3 | Dietary protein | CD | SG | 0.999–1.000 | 21.4–26.3 |
| 3 | Dietary protein | LP | MG | 0.999–1.000 | 3.0–14.0 |
| 3 | Dietary protein | LP | SG | 0.999–1.000 | 8.3–30.5 |
